# Analytical Performances of Nanostructured Gold Supported on Metal Oxide Sorbents for the Determination of Gaseous Mercury

**DOI:** 10.1155/2014/490291

**Published:** 2014-04-06

**Authors:** Julien Lusilao-Makiese, Emmanuel Tessier, David Amouroux, Ewa Cukrowska

**Affiliations:** ^1^School of Chemistry, Molecular Sciences Institute, University of the Witwatersrand, Private Bag Box X3, Wits, Johannesburg 2050, South Africa; ^2^Laboratoire de Chimie Analytique, Bio-Inorganique et Environnement (LCABIE-IPREM), UPPA, Hélioparc Pau-Pyrénées, avenue Pdt. Pierre Angot, 64053 Pau Cedex 9, France

## Abstract

Nanostructured gold supported TiO_2_, ZnO, and Al_2_O_3_ materials (1% w/w Au) were tested as sorbents for gaseous mercury (Hg) trapping and preconcentration. Their analytical performances were first compared with the one of traditional gold wool trap for the quantification of Hg standards injected into the argon flow followed by thermal desorption at 600°C and CVAFS detection. Good linearity and reproducibility were obtained, especially for Au/TiO_2_ material (*R* ^2^ = 0.995; slope: 1.39) in the volume range of 10 to 60 µL (132–778 pg Hg). This latter even showed a better performance compared to pure Au in the volume range of 10 to 100 µL (132–1329 pg Hg) when the carrier gas flow was increased from 60 to 100 mL min^−1^. The method detection limit (MDL) obtained with Au/TiO_2_ trap (0.10 pg Hg^0^ L^−1^) was suitable for total gaseous mercury (TGM) determination. Au/TiO_2_ was, therefore, used in trapping and determining TGM in collected air samples. TGM values in the samples ranged from 6 to 10 ng m^−3^. Similar results were obtained with the commercial gold-coated sand trap which showed an average TGM concentration of 7.8 ± 0.9 ng m^−3^.

## 1. Introduction

One of the most important environmental concerns of Hg is not only its toxicity but also its persistence and long-life in the atmosphere. Hg from point source emissions may remain localized in the environment or may be transported regionally and even globally [1]. Thus, simple, rapid, sensitive, and selective detection of atmospheric Hg is required when assessing the potential human health risk from an exposure.

Ambient air may contain considerable amounts of Hg, which are generally present in the elemental form (Hg^0^) [2, 3]. Sampling and the subsequent analysis of atmospheric Hg, which generally occurs at the femtogram level, is often made as TGM and is performed with highly sensitive detection methods combined with preconcentration techniques [4]. Gold and other precious metals are well known for their high efficiency in trapping Hg from the gas phase by amalgamation. Therefore, gold based collectors, which are used in many forms such as sand, wool, foil, wire, or deposits on different supports, play an important role in the preconcentration of Hg prior to detection [5–8].

In recent years, the catalytic properties of finely dispersed gold particles on oxide support materials have attracted much attention. Gold was rapidly recognized to be an extremely unique and highly active metal when prepared as supported nanoparticles [9, 10]. This is mainly due to the reduced dimensions of the gold particles and a strong interaction with the support [11, 12]. Thus, supports such as metal oxides are considered to be some of the materials adequate for preparing highly active supported gold catalysts [11]. There is a strong feeling amongst catalyst researchers that in a number of specific areas gold has a potential to be applied commercially, including in pollution control.

Thus, different types of nanostructured gold (or nanogold) materials have been recently tested in many research laboratories as Hg probes and chemosensors in natural waters [2, 13–17] or as oxidation catalysts for Hg^0^ in flue gases [18, 19].

In this paper, three nanogold supported metal oxides materials (Au/TiO_2_, Au/ZnO, and Au/Al_2_O_3_) were used for the sampling, preconcentration, and determination of gaseous Hg and their performances were compared to those of commercially available, namely, gold wool and gold-coated sand, sorbents.

## 2. Materials and Methods

Gaseous Hg was determined by double amalgamation cold vapor atomic fluorescence spectrometry (DA-CVAFS) [20]. Gaseous Hg species were trapped in a first column (sample trap) and desorbed to a second column (analytical trap) at 550–600°C. This latter usually contains pure gold that allows a more efficient thermal desorption (900°C) of the analyte to the detector. A transient signal was obtained with an increased signal/noise ratio. Detection limits were, therefore, lowered and the sensitivity was improved. The main advantages of the double amalgamation are found in the preconcentration of Hg at the second trap and in the reduction of interferences from water vapor and organic compounds during the first thermal desorption [21].

The calibration was done by injecting gaseous Hg standards directly to the analytical trap. The source of gaseous Hg was a drop of liquid Hg contained within a headspace bottle. The Hg drop was in equilibrium with the gaseous phase. A known volume of gas was collected with a microsyringe through a septum. The equilibrium concentration of Hg^0^ was, therefore, temperature dependent only and was measured with a thermocouple. The relationship between the vapor tension of Hg^0^ and the temperature is available in the literature [22]. Therefore, the amount of injected Hg^0^ could accurately be determined.

**Figure 1:**
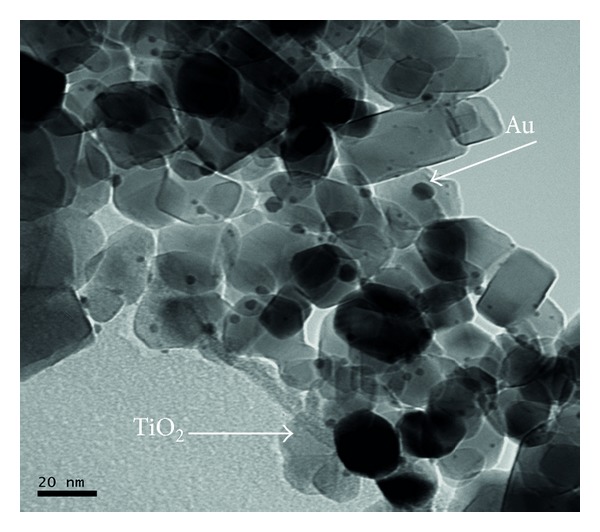
SEM image of gold particles dispersed on TiO_2_.

Hg^0^ standards were injected and “trapped” using commercial gold wool (Au), provided by the Laboratoire de Chimie Analytique, Bio-Inorganique et Environnement (LCABIE-IPREM, France), or nanogold supported on metal oxides (Au/TiO_2_, Au/ZnO, and Au/Al_2_O_3_). The nanogold sorbents pellets were obtained from Mintek (South Africa). The analytical sorbents consisted of 0.1 g of 1% (w/w) gold particles (<10 nm clusters) dispersed on metal oxides support (Figure 1). The sorbents were initially grinded, weighed accurately, and then held securely in a length of quartz tube, which was connected to a temperature controlled heater.

A coil of resistance wire surrounded the quartz tube and was resistively heated with precise temperature control during the analysis step until the coil began to glow dull red.

The trap was heated to release Hg, which was carried to the AFS detector (Tekran 2500, Canada), in a flow of pure argon (60 mL min^−1^). Analytical performances of nanogold materials were first evaluated by injecting Hg^0^ standards and by comparing the obtained instrument calibration lines to the one of commercial pure gold. A triplicate injection was performed for a given volume of standard for statistical purposes.

**Figure 2:**
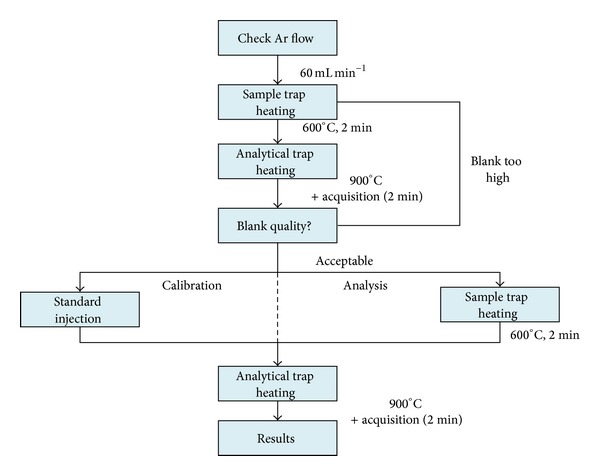
Analytical protocols for Hg standards calibration and TGM analysis.

The best performing analytical trap among the nanogold sorbents (i.e., Au/TiO_2_) was later used for the collection of gaseous samples and TGM analysis. The TGM determination was also carried out by double amalgamation cold vapor AFS (DA-CVAFS) and the performances of nanotraps were compared to those of commercial gold-coated sand traps. A summary of the analytical steps followed for both experiments is shown in Figure 2.

**Figure 3:**
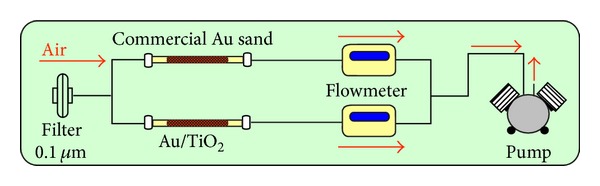
Schematic of sampling setup.

Simultaneous air collection for TGM measurements was done, as shown in Figure 3, with Au/TiO_2_ and gold-coated sand traps using a peristaltic pump (ASF THOMAS, Germany). The traps were connected in series to check on collection efficiency. The flow was controlled with a flowmeter (Bronkorst HiTech B.V. E-7000, Netherlands) in order to get an accurate measurement of the sample volume. The air sampling flow was 600 mL min^−1^ and a 0.1 *μ*m quartz filter (Whatman) was used to prevent the intrusion of dust and aerosols in the traps. Samples were collected at the ground floor (F1), first floor (F2), second floor (F3), and the roof (R) of the laboratory building.

For the collection of ambient air, a volume gradient was established in order to optimize the sampling time (i.e., the sample volume) and therefore to insure the samples representativity. Short collection times of 10, 20, and 40 min were used which corresponded to sample volumes of 6, 12, and 24 L (at a flow of 600 mL min^−1^). The air sampler was conditioned every day prior to sampling for stabilization and also for minimizing Hg adsorption on the walls. Sampling traps were later hermetically closed, stored in a double plastic bag, and analyzed as soon as possible.

It was necessary to perform a quality control before analysis and thus minimize analytical errors that could affect the Hg determination at such low level (memory effect, contamination, etc.). Therefore, analytical and field blanks were processed through the entire series of sampling and analytical steps which allowed us to determine the different method detection limits (MDL) and the methods limit of quantification (MLQ). Blanks were obtained by either passing UHP grade argon to the traps (analytical blank) or by replacing Whatman filters with the sampling sorbent in the sampling line (field blank). For the latter, the air passing through the sampling trap was, therefore, considered “Hg-free.” Field blanks traps were treated in the same way as for actual air samples collection. The resulting “blank” values were subtracted from the analytical results.

## 3. Results and Discussion

### 3.1. Analytical Performances

**Figure 4:**
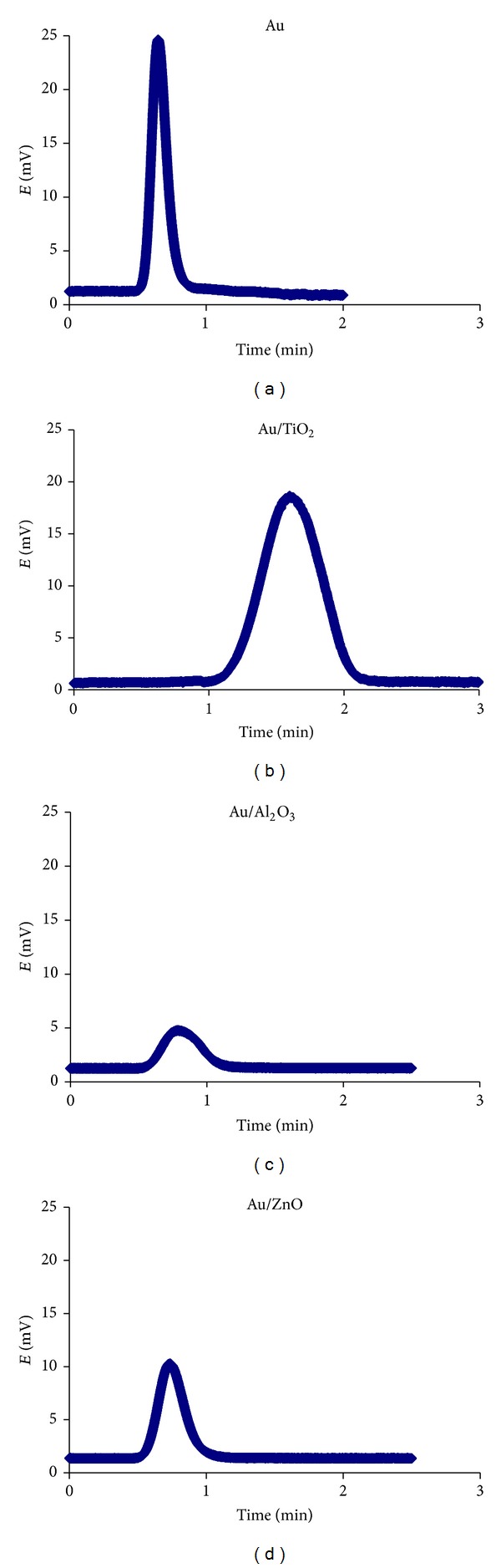
AFS chromatograms of 10 *μ*L Hg^0^ desorbed from different traps.

An example of signals obtained during analyses is presented in Figure 4.

**Figure 5:**
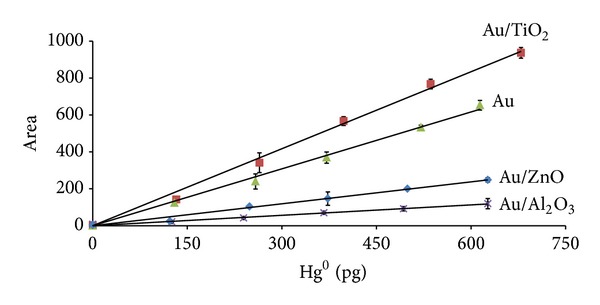
AFS calibrations of Hg^0^ standards at argon flow of 60 mL min^−1^.

A good linearity was obtained on the three nanogold materials, especially for Au/TiO_2_, in the volume range of 10–60 *μ*L Hg^0^, which corresponds to a concentration range of 131.8–777.5 pg Hg^0^ at an argon flow of 60 mL min^−1^ (Figure 5). In addition, signals obtained from triplicate injections of Hg^0^ standards when using Au/TiO_2_ as the analytical trap (Table 1) have demonstrated an excellent repeatability with RSD values always below 10%.

Other parameters such as retention time, number of theoretical plates, and full duration at half maximum (FDHM) height also have been studied and are presented in Table 2.

**Figure 6:**
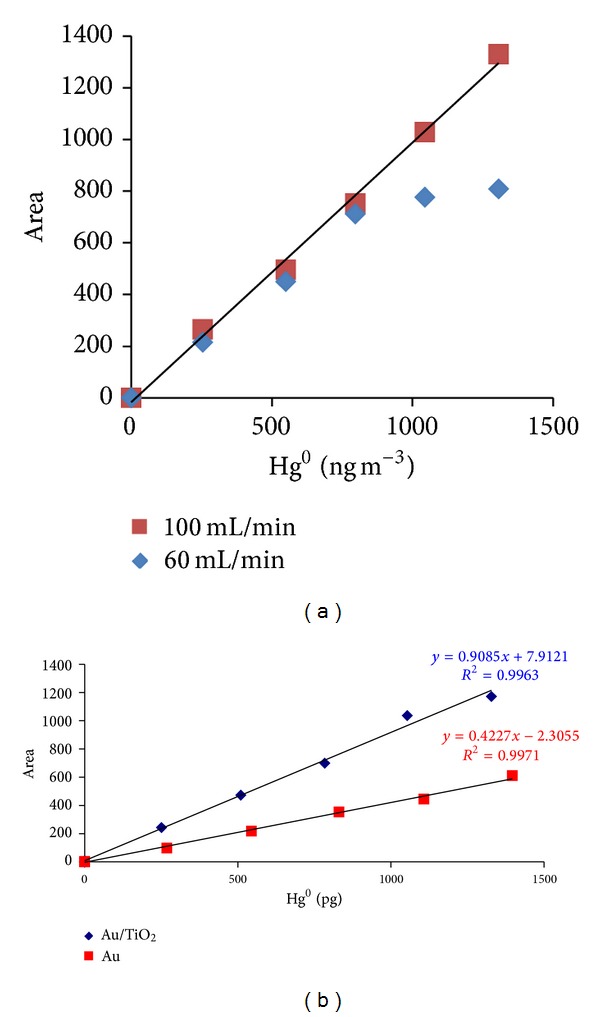
Calibrations obtained with Au-TiO_2_ at different Ar flows (a) and with Au and Au-TiO_2_ at Ar flow of 100 mL min^−1^ (b).

The linearity range of Au/TiO_2_ was even improved by increasing the carrier gas flow to 100 mL min^−1^. Calibrations with Au/TiO_2_ trap also exhibited a better slope, that is, better analytical response from the detector, under the above conditions compared to the one obtained with gold wool as shown in Figure 6.

It appears, based on the above results, that commercial Au wool presents the best characteristics: sharp peaks, best regression coefficient, and highest number of theoretical plates. Au wool was, therefore, considered to be the best analytical trap followed by Au/TiO_2_.

**Figure 7:**
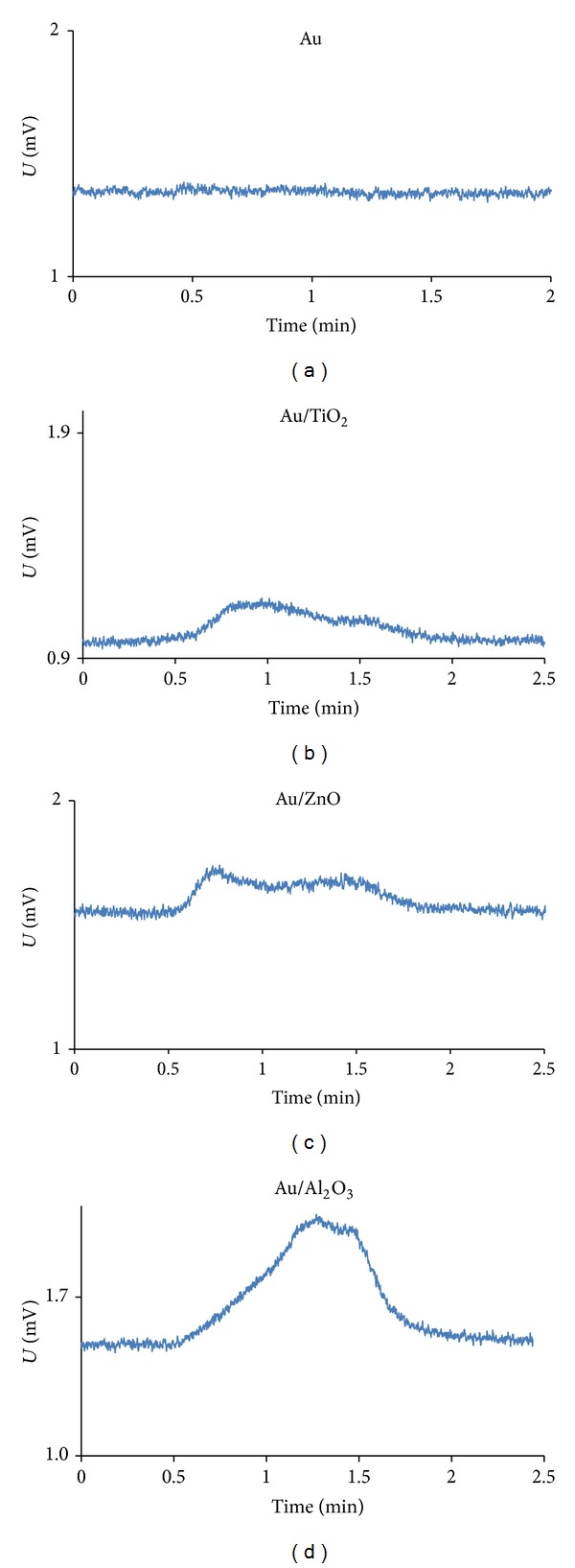
Examples of baseline obtained after the desorption of Hg from the different traps.

It is important to notice that, although chromatograms obtained with Au/ZnO and Au/Al_2_O_3_ had retention times similar to the one for Au wool, these materials showed lower linearity range compared to Au/TiO_2_ with lower slopes (Figure 5 and Table 2). They also have demonstrated poor baseline recovery after Hg desorption (Figure 7).

This implies a great retention of Hg vapor in the sorbents. Moreover, Au/Al_2_O_3_ materials exhibited hygroscopic properties (water retention) once exposed to the ambient air. Finally, Au/ZnO and Au/Al_2_O_3_ sorbents have shown different analytical responses (poor reproducibility) when the materials were roughly or finely ground. The difference in analytical performances observed between the nanosorbents could be attributed to the inner structure of the materials used, although this was not investigated in this study.

Numerous experimental and theoretical studies aimed at a better understanding of the properties of gold in the nanometer size regime have suggested that adsorption properties of gold catalysts are deeply influenced by the pore size and specific surface area of the material (see [12] and the references therein). Gold can be present as individual particles in the catalyst or can form agglomerations or clusters.

The size and the shape of the gold aggregates depend on the temperature and can be metastable once formed at low temperatures. These factors directly affect electronic, optical, and chemical characteristics of nanogold materials. In the case of the ultrathin oxide films, it was shown that the metal substrate lying underneath the film may affect the properties of gold particles, thus leading to an adsorption behavior that depends on the oxide thickness [12]. This could explain the fact that different adsorption capacities were observed with some sorbents (e.g., Au/ZnO), depending on the physical pretreatment.

Finally, studies have also shown that water strongly interacts with oxygen atoms in gold supported metal oxides to make a water-oxygen complex or hydroxyls [23–25], although Quiller and coworkers [26] have suggested that isolated stable hydroxyls may not be formed and could be more transient in character. The susceptibility of nanogold supported metal oxides materials to act as a Brønsted base or a nucleophilic base [27], due to the presence of oxygen atoms, could explain the “wetting” observed with the Au/Al_2_O_3_ sorbent. A deeper characterization of these materials is, therefore, of importance in order to improve their performance.

It can be seen from the above observations that Au/TiO_2_ has demonstrated better analytical performances compared to Au/Al_2_O_3_ and Au/ZnO. This is the reason why Au/TiO_2_ was selected to be tested as a sampling trap under real environmental conditions.

### 3.2. TGM Sampling and Determination

**Figure 8:**
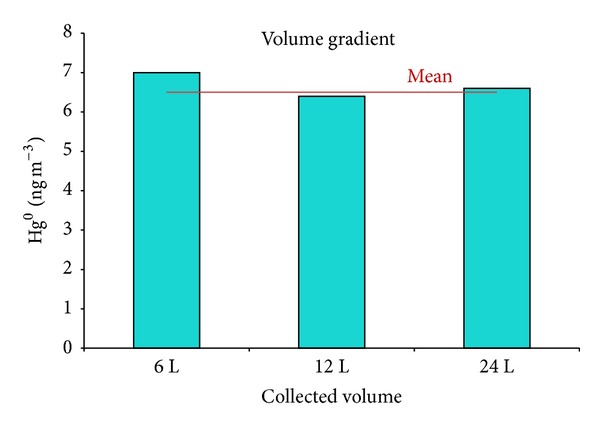
Concentrations of Hg^0^ as a function of sample volume.

As mentioned previously, in order to optimize the air sampling volume, a volume gradient was established using short collection times of 10, 20, and 40 min which corresponded to sample volumes of 6, 12, and 24 L at a flow of 600 mL min^−1^. The obtained Hg concentrations (mean value: 6.5 ± 0.5 ng m^−3^) were all beyond the method analytical performances (see Table 3). Due to sample variability caused by the air circulation in the building, the difference in Hg concentration (±8%) for the different sampling volumes was not significant (Figure 8). The optimized sampling time was, thus, chosen to be 20 min, that is, 12 L in volume.

The obtained methods detection limits (MDL) and methods limit of quantification (MLQ) are shown in Table 3.

Detection limit values were excellent and suitable for the detection of Hg^0^ at background level. It has to be recalled that TGM levels for background continental areas were reported to be in the range of 1.0 to 4.0 ng m^−3^ [28, 29].

Results of the TGM measurements performed on the collected air samples from the laboratory environment are presented in Table 4.

TGM concentrations measured with both Au-coated sand and Au/TiO_2_ traps were very similar with mean values of 7.8 ± 0.9 ng m^−3^ (range: 6.3–8.6 ng m^−3^) and 8.3 ± 2.3 ng m^−3^ (range: 6.0–10.1 ng m^−3^), respectively. Due to the range of TGM measured and the variability in samples (air recirculation in the building, climate changes, and activities in the laboratory), the obtained values were not considered to be significantly different. Moreover, statistical *t*-test and *F* test have been used to compare replicates measurements as well as the different standard deviations obtained with both gold-coated sand and Au/TiO_2_ traps. Results (Table 5) show that there is no significant difference between both replicates measurements and standard deviations for all the samples. Indoor (L1, L2, and L3) and outdoor (R) concentrations were almost similar, although the variability in R was higher than in L samples for both traps. This might be caused by changes in environmental conditions during sampling (wind direction and speed, air temperature, point sources, etc.).

It is well known that TGM exhibits an important diurnal variability with generally a maximum peak at midday [30]. The obtained average TGM values were within the range of reported mean TGM concentrations from urban areas [31, 32]. It is important to recall, here, that the collection efficiency of TGM using Au-coated quartz sand has been reported to be ≥95% [33]. Therefore, the closeness of TGM values measured with Au/TiO_2_ and the commercial Au-coated sand in the majority of the samples demonstrates the successful application of the nanogold trap under real environmental conditions.

Au/TiO_2_ has demonstrated a consistent response after being frequently used for about 3 months depending on storage conditions. A degradation of the adsorption efficiency (drop of signal intensity for a given concentration) was observed when the sorbents were exposed to the light for several hours.

## 4. Conclusions

This study has demonstrated the successful application of nanostructured gold supported in metal oxides materials for the trapping and preconcentration of Hg directly from the gaseous phase. Au/TiO_2_ has shown better analytical performances compared to Au/ZnO and Au/Al_2_O_3_, although it has also exhibited some retention problems. The superior performance of Au/TiO_2_ may relate to the grain morphology of TiO_2_, dispersion of gold particles, and the architecture of metal/oxide junctions.

The excellent MDL obtained with Au/TiO_2_ makes it suitable for background TGM determination. Environmental measurements of TGM with Au/TiO_2_ were similar to those obtained with traditional gold traps.

The development of an analytical procedure for TGM determination using Au/TiO_2_ sorbents was achieved in a performing and cost effective way compared to current methods since only 1% (w/w) of gold was required for the preparation of the materials. Finally, a deeper characterization of the inner structure of the studied materials is crucial to understand the different behaviors observed and to improve, where necessary, the performance of the materials.

## Figures and Tables

**Table 1:** AFS signals (area) obtained with Au/TiO_2 _trapusing 50 and 100 mL removable needle syringes (50 RN and 100 RN, resp.).

Volume of injected Hg^0^ (*μ*L)		Temperature (°C)		Corresponding Hg^0^ mass (pg)		Area	
50 RN	100 RN	50 RN	100 RN	50 RN	100 RN	50 RN	100 RN
5	10	19.3	19.7	62.1	128.5	38	145
5	10	19.6	19.8	63.7	129.6	36	140
5	10	19.6	19.9	63.7	130.7	33	140
10	20	19.3	20.1	124.3	265.7	78	362
10	20	19.4	20.4	125.3	272.4	76	320
10	20	19.5	20.4	126.4	272.4	84	350
20	40	19.6	19.9	254.9	522.7	211	750
20	40	19.6	20.2	254.9	535.9	232	754
20	40	19.7	20.5	257.0	549.4	229	797

**Table 2:** Analytical parameters of study materials.

Parameters	Au	Au/TiO_2_	Au/Al_2_O_3_	Au/ZnO
Retention time (min)	0.71 ± 0.05	0.83 ± 0.03	0.80 ± 0.01	0.73 ± 0.01
Slope (ua pg^−1^)	1.07	1.39	0.21	0.38
Regression coefficient	0.997	0.995	0.988	0.986
FDHM (min)	0.15 ± 0.01	0.43 ± 0.06	0.33 ± 0.03	0.20 ± 0.01
Theoretical plates	136 ± 7	36 ± 2	33 ± 5	69 ± 3

**Table 3:** Method analytical performances.

	*n*	MDL (ng m^−3^)*		MLQ (ng m^−3^)	
		Au	TiO_2_/Au	Au	TiO_2_/Au
DA/CVAFS	10	0.07	0.10	0.22	0.33
Sampling	5	0.15	0.19	0.52	0.62

*The MDL is calculated for a sample volume of 12 L.

**Table 4:** TGM in the laboratory ambient air where “Au” stands for gold-coated sand.

Sample	Hg (ng m^−3^)	
	Au	Au/TiO_2_
1st floor (L1)	7.0	7.8
	7.8	10.0
	8.5	12.5
Mean ± SD	7.8 ± 0.8	10.1 ± 2.4

2nd floor (L2)	6.8	6.8
	8.5	7.9
	9.8	8.9
Mean ± SD	8.4 ± 1.5	7.9 ± 1.1

3rd floor (L3)	4.8	5.1
	6.2	5.9
	7.9	7.0
Mean ± SD	6.3 ± 1.6	6.0 ± 1.0

Roof (R)	5.8	3.2
	8.3	9.2
	11.6	15.0
Mean ± SD	8.6 ± 2.9	9.1 ± 5.9

**Table 5:** Statistical results of TGM measurements (*t* and *F* were compared at 95% CI).

	*n*	Mean Hg (ng m^−3^)		SD (ng m^−3^)		*t*_calc_	*t*_table_	*F*_calc_	*F*_table_
		Au	Au/TiO_2_	Au	Au/TiO_2_				
L1	3	7.8	10.1	0.8	2.4	1.575	2.776	9.0	19.0
L2	3	8.4	7.9	1.5	1.1	0.466	2.776	1.9	19.0
L3	3	6.3	6	1.6	1	0.275	2.776	2.6	19.0
R	3	8.6	9.1	2.9	5.9	0.131	2.776	4.1	19.0
